# Integrated health and nutrition approaches to school feeding: maximising future human capital in Latin America and the Caribbean

**DOI:** 10.3389/fpubh.2024.1415172

**Published:** 2025-01-09

**Authors:** Laura M. Irizarry, Marie Evane Tamagnan, Carla Mejía, Heidi Kessler, Sarah Grundahl Kohnstamm, Giulia Baldi

**Affiliations:** ^1^Social Protection and Nutrition Unit, Programme Division, World Food Programme, Panama City, Panama; ^2^Inter-American Development Bank, Washington, DC, United States; ^3^Global Child Nutrition Foundation, Seattle, WA, United States

**Keywords:** school meals, Latin America and the Caribbean, human capital, integrated health and nutrition packages, social protection

## Abstract

Integrated health and nutrition packages in schools have been shown to be a cost-effective approach to support children’s well-being and academic achievement; yet few countries adequately invest in promoting such integration. School feeding programmes in Latin America are among the best-established, with some of the largest scale and coverage in the world. National School Meal programmes in Latin America and the Caribbean benefit over 80.3 million children and adolescents in the region. This paper seeks to determine the level of integration of complementary activities to school meal programmes in the region by examining their determinant factors: objectives, complementary services, governing regulatory frameworks, and monitoring and evaluation mechanisms. Our review reveals that although most governments offer school meals together with at least one complementary health and/or nutrition intervention, multisectoral commitment and investment is lacking. Under the premise that most school meal programmes in Latin America and the Caribbean have the potential to maximize their impact by providing integrated packages that meet children’s health and nutrition needs, we provide recommendations and raise considerations for the revision of programmatic guidance and policies moving forward. This analysis is relevant to countries internationally and calls for a comparable analysis to be carried out to promote a wide-reaching exchange on the integration of complementary activities to school feeding programmes globally.

## 1 Introduction

National school meal programmes in Latin America and the Caribbean (LAC)— which benefit more than 80.3 million children in the region (1, 2)— have great potential to affect their education, health, and nutrition status. One school meal a day can provide around a third of the daily caloric and micronutrient requirements for a healthy diet, that would otherwise not be available at home for the most food insecure households (3). An individual’s health and nutrition status are determinants of their ability to learn, and schools offer a unique space to influence both aspects (4). In the context of the multiple economic, political, and social challenges facing the region, school meals – together with other multisectoral activities – have enormous potential to contribute to human development. Achieving this promise requires an integrated approach to school meals that aims to contribute to the provision of balanced and nutritious diets, while enhancing other key activities essential to human capital development.

The strong body of evidence gathered by Bundy et al. (5) shows students’ health and nutrition are critical to their learning and educational achievement. Quality education- a key determinant to human capital- is not achievable in lieu of comprehensive health and nutrition strategies (5). It is well known that the availability of food in schools through school meal programmes increases educational coverage, reduces absenteeism, and can improve academic performance, particularly among the most vulnerable populations and girls (4, 6, 7). In addition to improving school attendance, Wang et al. (8) found that school meals also improve short term math and cognitive task performance among students in low- and middle-income countries. The findings by Wang et al. coincide with those of a 2019 study in Peru, which revealed that breakfast provided through the *Qali Warma* School Meals Programme has positive and significant short-term effects on cognitive test performance among children who do not eat breakfast at home (9). Investigations coincide in the crucial role of school meals in vulnerable and disaster areas and among the socially marginalized (6, 8, 9).

Complementary efforts to school meals can deepen their scope and impact. In contexts where there are deworming campaigns coupled with micronutrient supplementation, it is estimated that on average children obtain 2.5 more years of education (10). In turn, the delivery of fortified foods in schools can reduce anemia among adolescent girls by up to 20 percent (11). Handwashing alone can reduce absenteeism in schoolchildren by up to 61 percent by reducing the incidence of diarrheal and other hygiene-related diseases (12). In addition to the likely health and school achievement gains already mentioned, the potential financial returns of multisectoral school meal programmes in Latin America cannot be overlooked. School meal programmes are estimated to generate a return on investment of up to US$9 for every US$1 invested (1, 2, 13). A recent cost benefit analysis of school meal programmes in 14 countries, including Brazil, Chile, Ecuador, and Mexico, shows that investing in comprehensive, multisectoral school meals could amount to US$180 million in returns. In addition, the potential benefits for social protection are estimated at US$7 million and near US$23 million in returns for the agricultural economy. With an average feeding cost per child per year of US$105 for LAC potential gains in health and nutrition are from US$126 to US$335 per student, generating a cost benefit ratio of 2.6 (13). The latter is the highest cost benefit ratio, relative to other regions in the world evaluated by Verguet et al., such as US$54 in South Asia (1.6), or $US32 to US$140 in sub-Saharan Africa (2.3).

The school system is an exceptionally cost-effective platform for the delivery of an essential integrated package of health and nutrition services, including school meals, deworming, iron and folic acid supplementation, vision screening and oral health (1, 2). However only 11 percent of governments worldwide deliver the ideal fully integrated package of at least 6 interventions (14). When looking at the LAC region, the estimated potential returns of integrated school meal programmes to health, nutrition, and education activities, are far-reaching. This article, building upon prior exploration by the authors, seeks to explore the level of integration of complementary activities to school meal programmes in LAC by presenting a review of secondary data including, but not limited to, the objectives of school meals by country, complementary services offered in health and nutrition, complementary educational programmes and the regulatory frameworks that govern school meal programmes in the region (15). A country case-study approach was also undertaken to further explore the potential of multisectoral platforms. We conclude by drawing recommendations for future programmatic and policy reforms to school meal programmes that seek to maximize the human capital.

## 2 Assessing the integration of complementary activities to school meal programmes in LAC

### 2.1 Scope and main objectives of school meal programmes

School meal programmes in LAC have broad coverage during the initial and primary education levels and play an important role in the fight against hunger and malnutrition. According to a regional study carried out in 16 countries by the World Food Programme (WFP), 12 of the countries analyzed provide meals the preschool and primary levels (Bolivia, Brazil, Cuba, Dominican Republic, Ecuador, El Salvador, Guatemala, Honduras, Nicaragua, Panama, Paraguay, and Peru) (16). Only three countries (El Salvador, Cuba, and Brazil) reported providing school meals to students beyond primary school. The study indicates that, unlike school meals programmes elsewhere in the world, most of which have educational objectives as their main goal, in LAC health and nutrition are prioritized. In total, 12 of the 16 countries evaluated in this study (Brazil, Bolivia, Colombia, Cuba, Dominican Republic, El Salvador, Haiti, Honduras, Nicaragua, Panama, Paraguay and Peru) reported a specific or nutrition-sensitive component as their focus.

The findings of the 2017 WFP study coincide with those of the Global Survey of School Meal Programmes conducted by the Global Child Nutrition Foundation (GCNF), which last collected government-sourced data from countries for the 2020 school year. The survey captured information for the school year that began in 2020—a year that was at least partly, if not wholly, affected by the COVID-19 pandemic. In total, 139 countries—representing 81% of the world’s population—are included in the database. Of these, 125 countries had at least one large-scale school meal program, together providing information on 183 programs. Consisting of 11 sections, four sections in the survey contain national-level questions. The remaining seven sections contain program-level questions. For this analysis the sections on design and implementation and governance and leadership, were analyzed only for countries in Latin America and the Caribbean.

**Figure 1:**
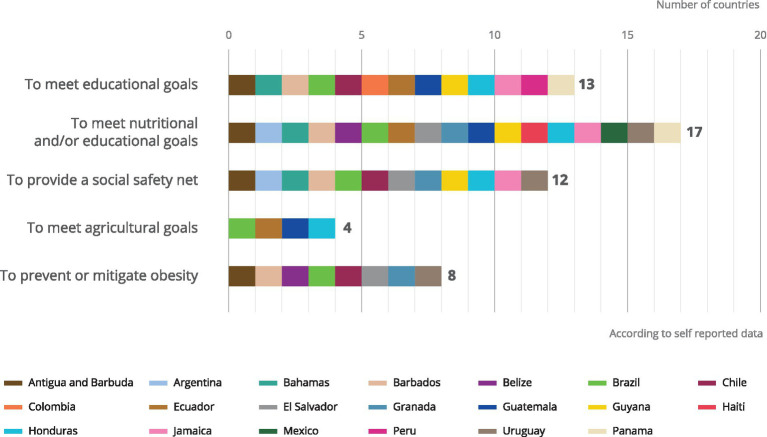
Objectives of school feeding.

Six possible main objectives of school feeding are considered in the survey (to meet educational goals, to provide a social safety net, to meet nutritional or health goals, to prevent or mitigate obesity, to meet agricultural goals, or other). Our region-specific analysis of that data finds that the main objective of school meal programmes in the region reported was to achieve nutritional and/or health goals, followed by educational goals, social protection goals, to prevent or mitigate obesity or to meet agricultural objectives (14) (Figure 1). Considering the evident problematic of the triple burden of malnutrition – chronic malnutrition, obesity, and overweight and micronutrient deficiencies – in the region, it is surprising that only eight countries report having specific objectives for obesity prevention and that only four report promoting increased access to diverse diets through agricultural objectives.

### 2.2 Activities implemented as part of school meal programmes

**Figure 2:**
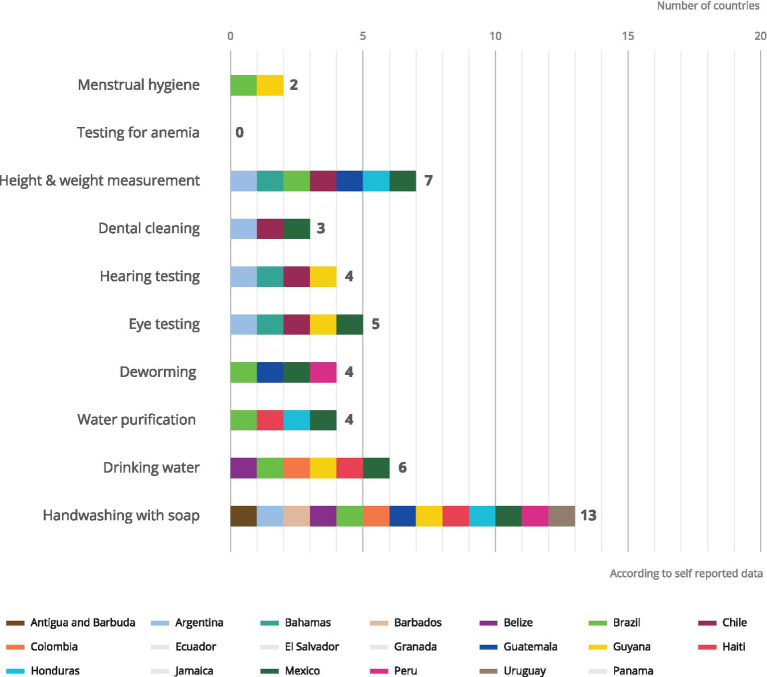
Complementary services offered in health.

Thirteen potential globally recognized health related complementary programs to school feeding options were also surveyed (handwashing with soap, height measurement, weight measurement, testing for anemia, deworming treatment, eye testing/eyeglasses distribution, hearing testing/treatment, dental cleaning/testing, menstrual hygiene, drinking water, water purification, other, none). The type of complementary activities to school meals implemented in Latin America and the Caribbean is consistent with the main objective of the programmes reported by country government focal points (to meet nutritional or health goals). Although it is true that a diverse set of activities are implemented in the region related to health promotion, the scope of their implementation is limited (Figure 2). As reported by countries, Water Sanitation and Hygiene (WASH) activities are implemented more frequently. However, even handwashing – which was most frequently implemented – was only reported by 13 of the 20 countries consulted by the GCNF. The measurement of height and weight was the second most reported activity in seven of the twenty countries. Other essential health strategies for academic achievement, such as visual and auditory evaluations, or menstrual hygiene management for girls and adolescents, are scarcely reported.

**Figure 3:**
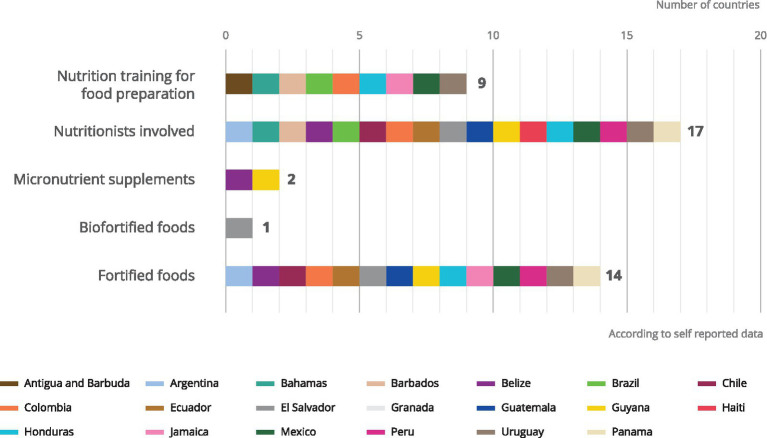
Complementary services offered in nutrition.

The GCNF assessed activities aimed at improving the nutritional quality of meals (nutrition training for food preparation, nutritionist involved, micronutrient supplements, biofortified foods, fortified foods) are implemented more frequently in LAC than in other regions. As shown in Figure 3, the majority (17) of feeding programmes in the region report having dedicated nutritionists as part of their workforce. Just under half of the countries consulted (9) offer nutrition training to those who cook and serve food to children. While 14 out of the 20 countries surveyed report providing fortified foods through school meals programmes, these are hardly ever implemented alongside complementary strategies necessary to amplify the impact of the delivery of micronutrients. For example, treatment of parasitic diseases is essential for good health and to harness the full benefits of micronutrient supplementation and fortification (17, 18). Only four countries reported deworming efforts as part of their school meal programmes.

**Figure 4:**
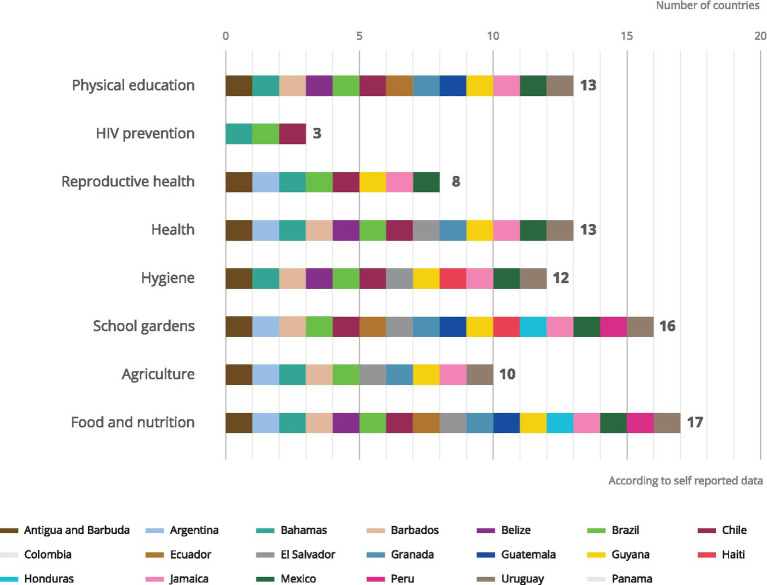
Complementary education programmes implemented as part of school feeding programmes.

Eight potential complementary education programmes implemented alongside school meal programmes were evaluated 7hysical education, HIV prevention, reproductive health, health, hygiene, school gardens, agriculture, food, and nutrition. In the LAC region, the most implemented were related to food, health, nutrition and physical activity (Figure 4). At least 50 percent of countries reported implementing some form of a complementary education strategy. The frequency in which the topics of food and nutrition, school gardens, health, and physical education are addressed in the form of complementary educational strategies through school meals is in line with the objectives of the programmes and the commitment to improving health and nutrition.

### 2.3 Institutional and regulatory frameworks

**Figure 5:**
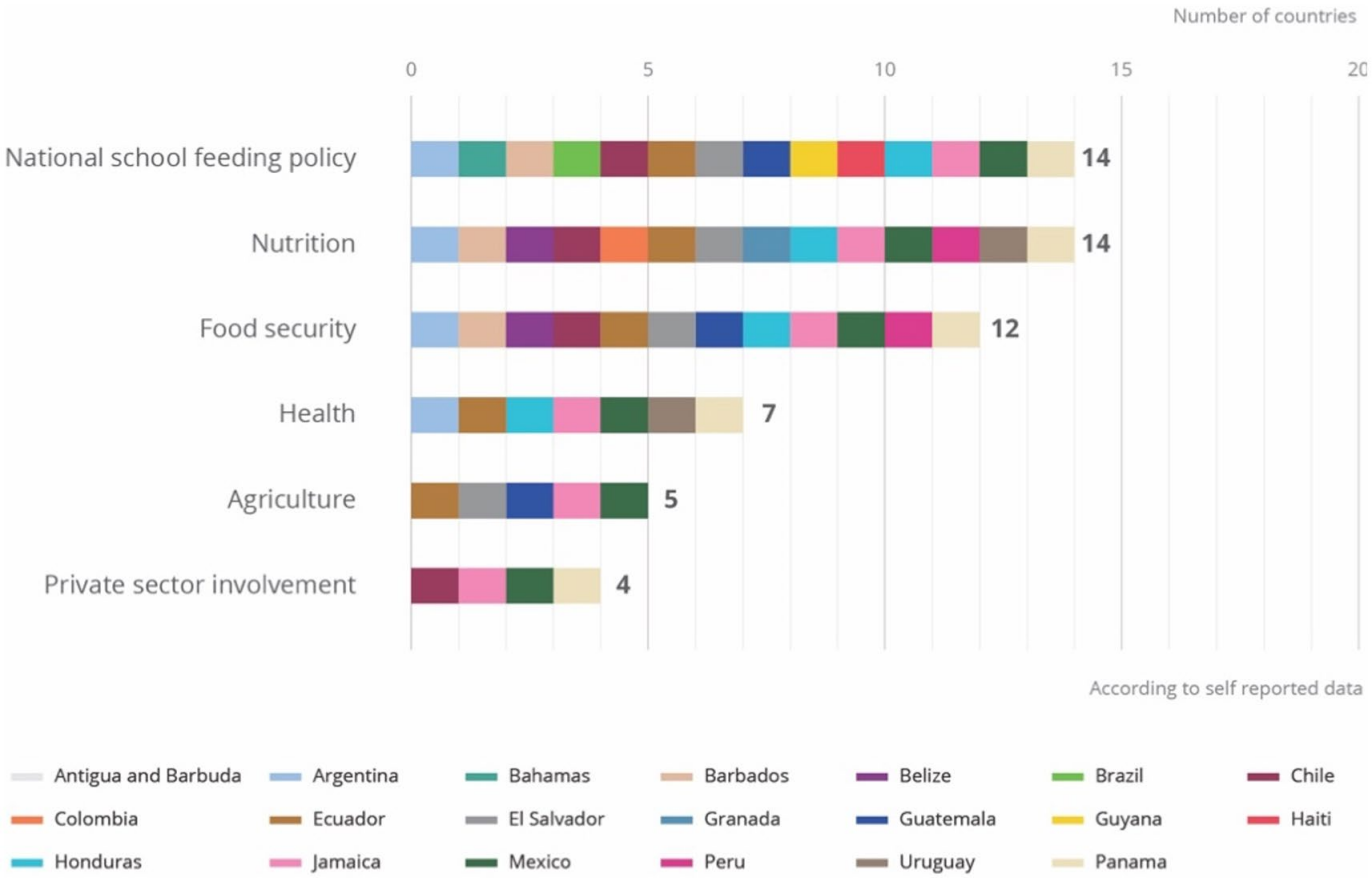
Regulatory frameworks in school feeding.

A careful examination of the laws, public policies and national standards governing school meal programmes provide valuable insight into programmatic priorities. According to the government-reported information in the Global Survey of School Meal Programmes, 70 percent of the programmes surveyed in the region reported having a school meals law, public policy, or national standards in place; four potential topics where surveyed (national school feeding policy, nutrition, health, food safety, agriculture). The same proportion, or 14 out of 20 countries, report having a policy related to school meals specific to nutrition and just over half the countries report having a food safety policy. Only a few countries have any related health policy (7), or agricultural policy (5) linked to school meals (Figure 5).

The implementation of legal instruments that strengthen and ensure the promotion of food and nutrition security is an important resource for placing responsibility on the State and civil society; it is also central in setting food and nutrition as a priority. Brazil, a global reference for school feeding for almost 70 years, has been successful in setting food security and nutrition as a national priority through two critical actions: the progressive institutionalization of the school feeding through the creation of public policies and legal frameworks, but most notably through civil society mobilization (37). Since 2009, Brazilian law regulates the coverage and management of the programme including delivery time, type of food, the form of participation, public procurement, the monitoring and evaluation system, and school gardens as a pedagogical tool, among others. Based on the Brazil experience, other countries in the region have recently adopted frameworks for sustainable school feeding including Guatemala (2017), Honduras (2016) and El Salvador, which initiated the process in 2021.

### 2.4 Monitoring and evaluation

Despite the ample scope of school meal programs and the various strategies and activities that are integrated into them, there are few adequate monitoring mechanisms or precise indicators to measure their impact (19). Some cases do not report national monitoring and evaluation systems that inform policy makers or programme managers on the implementation quality and effectiveness of the school meals programmes, and their various strategies. Hence, it is difficult to take timely corrective actions. Several countries have done evaluation exercises and have developed indicators for acceptability, scope of assistance and well-being, among others. Yet, most existing mechanisms being implemented are insufficient to measure the performance and real cost of programmes or robust enough to respond in situations where rapid action, coordination or innovation is required (20).

Chile, where a comprehensive country specific evaluation of the school meals programme was recently published, offers a relevant example of the implications of assessment techniques on programme quality. Important determinants such as relevance, coherence, efficiency, effectiveness, sustainability, and evaluability were addressed as part of the evaluation (21, 22). A proposal for programme improvement resulted from this analysis, which demonstrated the need for a redesign of public policy to overcome gaps in quality, coverage, and efficiency.

Existing policy analysis tools, such as the Systems Approach for Better Education Results (SABER) offer comprehensive instruments to identify priority areas for strengthening education systems (23). The tool assesses 10 related topics that range from early childhood development to workforce development, including health and school feeding. The SABER-School Health and School Feeding documents that analyze school health programs for children of school age and SABER-SHSF diagnostic tools can be used to determine country’s progress in implementing each indicator and provide a snapshot of the developmental status of school health policy in the country. Presently WFP, which is the largest humanitarian provider of school meals worldwide, requires that all countries that intend to implement school feeding programs with WFP support must undertake a SABER analysis of school feeding. Its completion is also a prerequisite for any World Bank grant to be awarded related to education, positioning it as a useful tool that is readily available to countries.

## 3 Way forward and recommendations

The mandatory nature of primary education in LAC guarantees that school meal programmes are positioned as an ideal facilitator of programmes’ integration. Under the premise that children and adolescents should reach their full potential and are guaranteed the right to healthy food, to education and to protection, strengthening school meal programmes should be a priority. The information available across the region suggests that school meal programmes when integrated with health and nutrition initiatives have the potential of optimizing performance and could offer greater impact. This is reflected in the variety of complementary activities that exist – covering a range of activities related to a greater extent to health and nutrition, and agriculture and food production. Although the intention to foster better integration are authentic, they are insufficient, thereby leaving tangible results unrealized or unknown for students and for society. Still, the region offers important lessons to draw from in countries with a high level of activity integration, such as Chile.

### 3.1 Designed for success: the Chilean integrated model

Recent evaluations of the *Programa de Alimentación* in Chile confirm its role in combating malnutrition by providing adequate nutrients for students (21). It has several cross-sector regulatory frameworks and cross-sectoral objectives (14). Among its programmatic objectives, it seeks to fulfil educational objectives, offer a social safety net, and prevent malnutrition. Regarding regulatory matters, the program integrates public policy on school meals and specific policies on nutrition, food security and private sector involvement. Yet, what sets the program apart is that school meals are part of an institutional programmatic offer that is focused on promoting access to health and nutrition, providing anthropometry, hearing tests and visual and dental controls. In addition, complementary education programmes in food and nutrition, school gardens, hygiene, school health and recreational activities are delivered. The program also offers fortified foods, has the support of nutritionists in its planning and implementation, and aims to ensure children are well nourished at school, while preventing obesity. The program has an additional characteristic that differentiates it from other programs in the region: it provides students meals from nursery school to higher education, delivering food that meets the needs for each developmental stage.

The challenge of implementing regulatory frameworks to support programmes and monitoring and evaluation systems to oversee them of the most pressing identified by the authors. If programmes are not consistently monitored and evaluated their efficacy and efficiency cannot be adequately assessed. This in turn, leaves program managers and policy makers unable to decide how or whether to improve these programmes or the relevant policy instrument. It is recommended to consistently apply monitoring and evaluation techniques to encourage quality optimization and continuous learning. Brazil offers a promising example in the region for other countries to draw lessons from.

### 3.2 Brazil: the role of civil society in program monitoring and accountability

The Brazilian National School Feeding Programme (PNAE) one of the oldest and wide-reaching programs in the world offers an important example of advancement and innovation over its 45-year history. Formally established in 1979, but it’s groundwork dating back to 1940, the program has matured over the decades, providing important lessons for the region particularly with regards to monitoring and the role of civil society oversight (24). The PNAE is monitored and supervised directly by civil society through School Feeding Councils (Conselho de Alimentação Escolar – CAE) and institutionally through FNDE and by control bodies such as the Federal Court of Accounts (Tribunal de Contas da União – TCU), the Comptroller General of the Union (Controladoria Geral da União – CGU), and the Public Prosecutor’s Office. Monitoring focuses mainly in the adequate use of the programme’s financial resources, but also involves nutritional and cultural adequacy of menus, quality of meals, efficiency of local management, and compliance with central PNAE regulations. Accountability for the use of public resources is a constitutional duty of public managers. A digital tool was created in 2019 to make the process accessible and transparent; the *ePNAE* mobile application^1^ was developed by the National School Feeding Programme to allow parents, students, teachers, nutritionists, school food managers and other stakeholders to monitor and evaluate the school meals offered in public schools throughout the country. While challenges can arise, the PNAE is a successful example of mechanisms for adequate and timely monitoring in every territory through multisectoral collaboration, with emphasis on the civil society.

### 3.3 The case for promoting integrated health and nutrition packages through school feeding

The current context of economic slowdown, socio-political instability, migration crises, global warming, demographic, epidemiological and nutrition transitions, and the aftermath of the COVID-19 pandemic outcomes, threaten to reverse the results achieved in advancing the situation of health and nutrition in the region over decades. In parallel, the dramatic increase in the cost of food leaves access to adequate and nutritious diets out of reach for 22.5% of the LAC population (25). These socio-economic issues have caused the region’s epidemiology of malnutrition to change, bringing new challenges to LAC countries. While undernutrition remains unresolved; overweight and obesity prevalence continue to increase, affecting large proportions of the population throughout the life cycle, particularly children (26). Childhood obesity is associated with an increased likelihood of obesity in adulthood, disability, and premature death. It is the main risk factor for non-communicable diseases, which are the leading cause of death in the region (27).

There is strong evidence that the in-school environment shapes diets and therefore, the nutritional status among children (28, 29). Thus, school meals programs integrated with policy instruments and programs that simultaneously reduce the risk or burden of both undernutrition and overweight/obesity are critical for adequate prevention and management of these conditions. Moreover, regulation of school environments and food sold in schools has proven to be effective in multiple contexts (30, 31) if oversight regarding compliance with regulatory measures is established and the community participates in the fulfillment of public policies especially about marketing and advertising (32). To foster availability and consumption of diverse fresh foods at school, policy frameworks that require procurement from local farmers have been successfully promoted in countries in the region such as Mexico and Brazil (33, 34). However, implementation of these programmes has been possible only when coordinated with sectors including agriculture and WASH and adequate infrastructure is sustainably financed. It is recommended to continue fostering inter-sectoral health, nutrition, and school meals programmes as they have the potential to contribute significantly to fill the food security and malnutrition gap for millions of children and adolescents in the region. Furthermore, enhancing their quality and reach is urgent.

The latter is not an easy feat; it requires concerned actions at the regional and country levels, not only to advocate on the programmes’ value – what is already well known-but also to reconceptualize school meals from a multisectoral perspective that promotes comprehensive policies and financial commitments for their implementation and sustainability over time. As reported by countries in the GCNF (14), inadequate resources and unpredictable funding was a central barrier to programme oversight, planning, implementation, and development. The mismanagement of resources through programmes was also stressed as a recurring problematic. Global and regional initiatives, including the School Meals Coalition – a global platform gathering 106 countries worldwide (18 in LAC) with the objective that every child receives a healthy, nutritious daily meal in school by 2030—and the Sustainable School Feeding Network—a network promoted by the Government of Brazil to jointly and broadly create solutions to the challenges of school feeding in Latin America and the Caribbean—advocate that programmes are implemented alongside an integrated health and nutrition package of interventions and advocated in favor of public financing for them. These collective efforts, which also promote capacity building, the exchange of experiences and information and a cooperative dialogue, should be capitalized upon by countries seeking to strengthen their programmes and to overcome policy and programmatic challenges (35, 36).

The Global Survey of School Meal Programs, which was the basis for the analysis, is the first successful attempt to elicit standardized information about school meal programs and associated activities directly from government sources, from all regions of the world, regularly. However, several limitations should be acknowledged. The Global Survey data referred to in this paper is based on the 2020 school year (the latest available data at publication), during the COVID-19 pandemic, and therefore may capture effects of the global crises which may be temporary or permanent. The Global Survey is completed by a government appointed focal point. Even though respondents were asked to complete the survey to the best of their abilities, the data may be subject to bias or inaccuracy. In addition, the survey was not completed by all countries in the region. Fostering the completion of the survey by all countries on a regular basis is recommended, to enable monitoring school meal programme trends over time. This will allow decision makers to direct resources to areas of greatest need to support the implementation of high-quality school meal programmes that incorporate complementary health and nutrition packages.

The scaling up of health and nutrition efforts also implies considerations of potential tradeoffs in terms of cost and efficiency. This is probably one of the reasons why, although evidence indicates that adolescents, especially girls, are a critical group for school-based health and nutrition interventions, current programming rarely covers this population group. The reasoning to support integrated packages that meet all of children’s health and nutrition needs so they can learn and thrive is irrefutable: not investing in a healthy and educated population compromises the sustainable development of countries. Latin America and the Caribbean is already ahead, relative to other regions, in complementing school meals with other activities or fully integrating them as part of the school meals programme (14). The timing is therefore right to consider the next steps that would position the region as the pioneer of a new era in public health, with schools at the center.

## Footnotes

^1^ https://www.gov.br/fnde/pt-br/acesso-a-informacao/acoes-e-programas/programas/pnae/controle-social-cae/e-pnae
